# α-Borophene Nanoribbons: Edge-Dependent Metallic and Magnetic Properties for Low-Dimensional Nanoelectronics

**DOI:** 10.3390/molecules30214177

**Published:** 2025-10-24

**Authors:** Subrata Rakshit, Favian Sun, Nevill Gonzalez Szwacki, Boris I. Yakobson

**Affiliations:** 1 Faculty of Physics, University of Warsaw, Pasteura 5, PL-02093 Warszawa, Poland; srakshit@fuw.edu.pl (S.R.); gonz@fuw.edu.pl (N.G.S.); 2 Department of Materials Science and NanoEngineering, Rice University, Houston, TX 77005, USA; fs67@rice.edu

**Keywords:** borophene nanoribbons, DFT, electronic properties, electronic transport

## Abstract

We present a comprehensive first-principles study of nanoribbons made from the α-borophene sheet. This study looks at how edge shape, ribbon width, and magnetic ordering affect their structural, electronic, and transport properties. Ribbons cut along armchair (ac) and zigzag (zz) directions with various edge designs—armchair (a), single (s), and double (d) chains—are all stable. The double chain “dd” edges have the highest binding energies and the lowest edge energies, which aligns with near-bulk coordination. Our analysis of electronic structure and ballistic transport shows strong metallic characteristics in almost all configurations. Only the narrowest “3-ad” ribbon shows a small energy gap that disappears as the width increases. Zigzag ribbons (“zz”) display edge magnetism that depends on width, changing from non-magnetic to antiferromagnetic and finally to ferromagnetic states. Their spin-resolved transmission demonstrates clear spin filtering with polarization exceeding about 40%. Edge passivation affects these properties: hydrogen and fluorine reduce the “zz” edge magnetic moments and spin transport, while oxygen maintains finite magnetism. Near the Fermi level, many ribbons allow for multiple conducting channels. This feature supports low-resistance charge flow even for widths below 10 nm, while higher-energy transmission shows greater dependence on width. These findings position α-borophene nanoribbons as promising one-dimensional components for nanoelectronic connections and spintronic devices, combining high stability, adjustable edge magnetism, and strong metallic conduction.

## 1. Introduction

Low-dimensional materials continue to reveal unique physical phenomena and hold promise for nanoscale electronics, energy applications, and sensing technologies. Among them, boron—a neighbor of carbon in the periodic table—has emerged as an especially versatile element for constructing low-dimensional nanostructures due to its electron deficiency, multicenter bonding, and polymorphism [1,2]. The possibility of boron-based analogs to carbon structures, including fullerenes [3,4], nanotubes [5], and two-dimensional (2D) borophene sheets [6], has opened a rich frontier in boron nanoscience [7,8].

Borophene, a single-atom-thick sheet of boron, has been synthesized on metallic substrates such as Ag(111) and Ag(110) using physical vapor deposition methods under ultrahigh vacuum conditions [9,10]. Unlike graphene, borophene is not a single-phase material but comprises several polymorphic phases, including *α*, *β*_12_, *χ*_3_, and *δ*_6_, characterized by a mixture of triangular and hexagonal motifs [11]. These polymorphs exhibit anisotropic bonding networks, high flexibility, and metallic behavior, making borophene and its derivatives highly attractive for applications in flexible electronics, batteries, and energy storage [12,13].

A natural variety of borophene’s two-dimensional (2D) structure is the formation of borophene nanoribbons (BNRs, an analog of extensively studied GNR–graphene nanoribbon [14,15]), which are quasi-one-dimensional (1D) strips characterized by controlled edge morphology and tunable widths. Conceptually, BNRs can be constructed by cutting a borophene sheet along defined crystallographic directions, resulting in edge-dependent properties [16]. Previous first-principles calculations show that hydrogen passivation of zigzag boron nanoribbon (BNRs) edges stabilizes the structures and opens semiconducting gaps that oscillate with ribbon width [17]. Transport calculations further indicate a pronounced width dependence in BNRs devices, with wider ribbons delivering substantially higher conductance [18]. In terms of structural origin, nanoribbons obtained from the α-boron sheet are energetically favored over those from triangular or reconstructed sheets, and their stability increases with width; while most BNRs are metallic, semiconducting cases have also been reported [19]. Complementary NEGF/TB calculations on α-BNRs FM/NM/FM junctions predict pure spin currents and spin voltages under thermal gradients, with perfect spin filtering and diode-like response at low temperatures. The spin figure of merit grows with stronger exchange fields and decreases with ribbon width, underscoring the promise of narrow α-BNRs for spin-thermoelectric devices [20]. Recent DFT studies indicate that nitrogen termination of zigzag α-BNRs edges stabilizes the ribbons and can open a band gap. Subsequent Cr or Mn doping induces robust ferromagnetism with Curie temperatures above 700 K, resulting in a magnetic metal for Cr and a diluted magnetic semiconductor for Mn [21]. 1D boron chains undergo a transition from metallic to semiconductor, driven by the formation of spin density waves [22]. Recent advances in topological and mechanochemical design have enabled the fabrication of complex ribbon architectures, including rectangular and bi-trapezium-shaped motifs derived from *β*_12_ and *χ*_3_ borophene phase [23]. These designs provide powerful handles for tuning the band structure, electronic transport and bonding networks of BNRs [24,25]. Furthermore, advanced many-body computational methods such as thermally assisted-occupation density functional theory (TAO-DFT) have revealed that large BNRs exhibit pronounced size-dependent electronic properties and strong static correlation effects due to their inherent multiradical character [26]. Previous studies have shown that the presence of double- or triple-chain edge structures enhances the stability of borophene nanoribbons [27].

Experimental efforts have succeeded in synthesizing BNRs with widths in the range of 10–100 nm and lengths up to several micrometers using various vapor-phase techniques, including chemical vapor deposition and magnetron sputtering [28,29]. Structural analyses indicate that these nanoribbons may adopt tetragonal crystalline cores with amorphous surface layers. Meanwhile, scanning tunneling microscopy (STM) and first-principles simulations have confirmed the atomic-scale periodicity and polymorphism in epitaxial BNRs grown on Ag surface [30].

In the present work, we investigate boron nanoribbons derived from the α-sheet borophene polymorph, characterized by a hexagonal hole density of *η* = 1/9, where *η* denotes the ratio of hexagonal vacancies to total atomic sites in the ideal triangular lattice. This particular configuration is chosen because both theoretical predictions and experimental observations identify α-borophene (*η* = 1/9) as one of the most stable borophene phases [1,6]. We construct nanoribbons by trimming the α-sheet along two principal crystallographic directions: armchair (ac) and zigzag (zz). For the ac-oriented nanoribbons, we design multiple edge configurations by combining distinct motifs—namely “a” (armchair chain), “s” (single chain), and “d” (double chain)—to explore the impact of edge geometry on electronic behavior. To further investigate the influence of passivation, borophene nanoribbons (BNRs) edges are passivated with hydrogen (H), oxygen (O), and fluorine (F). Using density functional theory (DFT) calculations, we systematically examine the structural stability, binding energies, electronic band structures, magnetic properties, and ballistic transport characteristics of selected ribbon types. Our results demonstrate the persistence of metallicity across a wide range of edge configurations and reveal clear structure–property correlations. These findings offer valuable insight into the tunability of α-borophene nanoribbons and their potential as functional one-dimensional components in future nanoelectronic and spintronic devices.

## 2. Computational Methods

All first-principles calculations were carried out within the framework of DFT using the Perdew–Burke–Ernzerhof (PBE) exchange–correlation functional and norm-conserving pseudopotentials from the PseudoDojo library [31], as implemented in the Quantum ESPRESSO (QE) package (v7.2) [32]. The plane-wave kinetic energy cutoff was set to 80 Ry. Structural relaxations were performed by optimizing both atomic positions and cell parameters until the forces on all atoms were below 0.3 meV/Å and the total energy change per self-consistent cycle was less than 10^−5^ Ry. Spin polarization effects were included, and the systems were sampled using a Γ-centered 1 × 16 × 1 Monkhorst–Pack k-point mesh along the periodic direction. To simulate isolated 1D systems, we employed vacuum regions of 12 Å. The electronic structures were obtained on dense k-point grids of 1 × 48 × 1 along periodic direction to ensure accurate interpolation of the electronic bands. Visualization of atomic structures was performed using the VESTA software (v3.5.8) [33].

Ballistic transport calculations were performed using the TranSIESTA (v5.2.1) [34,35] code under the Non-Equilibrium Green’s Function (NEGF) formalism. Without relaxing the structures in SIESTA, the transmission steps have good agreement with the band structures computed in QUANTUM ESPRESSO. The electrodes were sampled using 101 k-points in the periodic direction, while the scattering region was sampled with only the gamma point. Eigenchannel wavefunctions are computed using the Inelastica package (v1.3.7) [36,37]. Current under a finite voltage was computed using the Landauer–Büttiker equation,

(1) $$I(V)=\frac{e}{h}\int \;\;T(E,V)[f(E,\mu +V/2)-f(E,\mu -V/2)]dE$$

where T(E,V) is the mode-weighted transmission value under a bias V, f(E,μ) is the Fermi distribution function at a chemical potential μ, e is the charge of an electron, and h is Planck’s constant.

## 3. Results

**Figure 1:**
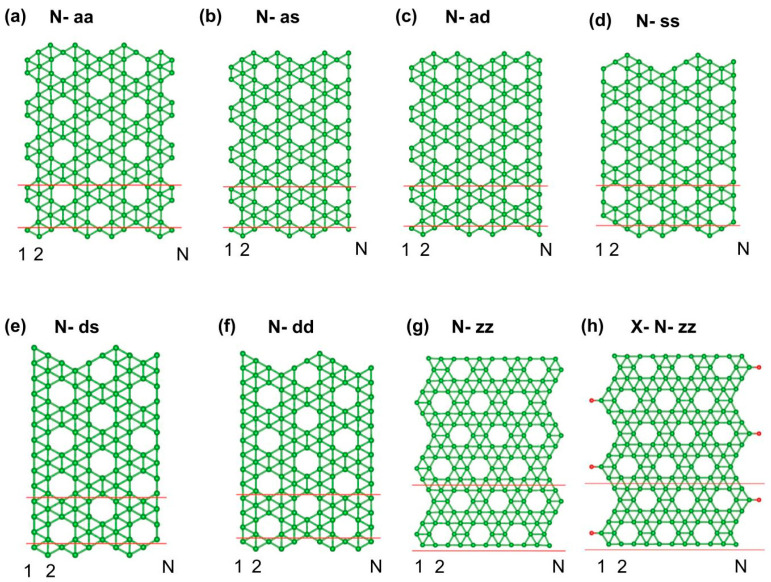
(**a**) N-aa (both edges are armchair chain), (**b**) N-as (one edge armchair chain and another edge single chain), (**c**) N-ad (one edge armchair chain and another edge double chain), (**d**) N-ss (both edges are single chain), (**e**) N-ds (one edge double chain and another edge single chain), (**f**) N-dd (double chain at both edges), (**g**) N-zz, and (**h**) X-N-zz (edges are passivated with X, where X = H-, O-, F-, shown as red dots). The red lines delineate the unit cell.

We classify the borophene nanoribbons (BNRs) into seven distinct categories (as shown in Figure 1): six derived from the armchair (ac) direction and one from the zigzag (zz) direction. The adopted nomenclature is defined as follows: “aa” indicates ribbons whose edges both consist of armchair chains; “as” denotes ribbons with one armchair edge and one single chain edge; “ad” represents ribbons with one armchair edge and one double chain edge. Similarly, “ss”, “ds”, and “dd” denote ribbons whose edges consist, respectively, of single–single, double–single, and double–double chain combinations. The “zz” refers to ribbons oriented along the zigzag direction. The parameter N specifies the number of chains spanning the ribbon width. In addition to the pristine BNRs, the “zz’’ BNRs are further investigated by edge passivation using hydrogen (H), oxygen (O), and fluorine (F) atoms on their structural and magnetic properties. The “zz” BNRs are specifically chosen because they exhibit magnetism at the zigzag edges. This makes it an ideal candidate to explore how different passivating atoms influence the stability and edge magnetism, in contrast to non-magnetic configurations such as “dd” BNRs.

**Figure 2:**
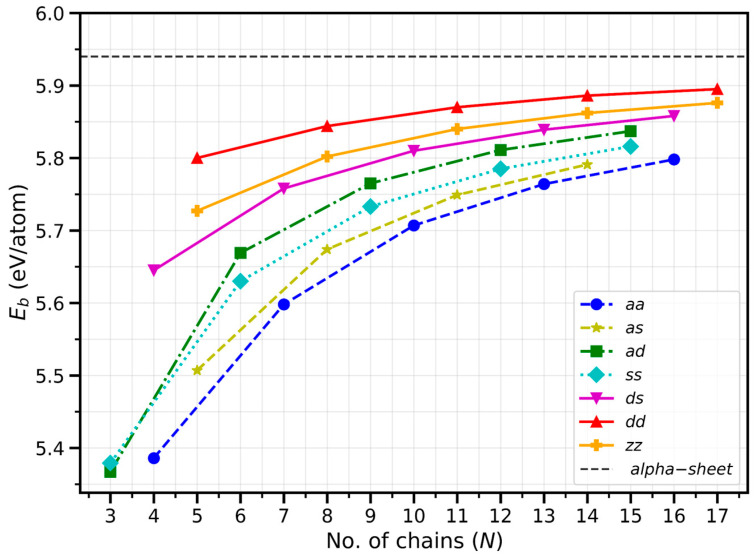
Binding energy vs. the number of boron chains. The value for the α-borophene phase is $E_{b}$ = 5.94 eV/atom. All follow closely an analytical relation $E\;=\;E_{b}\;-\;\varepsilon /N$, where ε values reflect the energies of the edges.

Figure 2 provides a systematic illustration of how the binding energy $E_{b}$ varies with nanoribbon width and edge shape. The binding energy is used to evaluate the stability of the BNRs. It is calculated using the formula: $E_{b}=(n\times E_{n}\;-\;E)/n$, where $E_{b}\;$ is the binding energy per atom, n is the number of atoms, $E_{n}$ is the energy of one single boron atom, E  is the energy of one BNR. A higher (more positive) binding energy indicates greater stability of the BNRs. A general and consistent trend observed across all edge types is that the binding energy $E_{b}$ increases monotonically with ribbon width (i.e., with the number of chains N). This indicates that wider ribbons are more energetically favorable. For instance, in “aa”, $E_{b}$ increases from 5.386 eV/atom for “4-aa” to 5.798 eV/atom for “16-aa”. Similar monotonic increase is evident for “ss” (from 5.379 to 5.816 eV/atom), “ds” (from 5.645 to 5.858 eV/atom), and “dd” (from 5.80 to 5.895 eV/atom) edge families. This trend is the same for all types of edges, reflecting a general tendency toward bulk-like stability as the ribbon width increases and edge effects weaken, supporting the potential feasibility of synthesizing such nanoribbons experimentally.

While width plays an important role, the edge structure shows an even more pronounced effect on the stability. Among the seven categories of BNRs considered, the double chain edge (“dd”) consistently exhibits the highest binding energy for any given width. For example, at comparable widths (~12 Å), binding energy $E_{b}$ = 5.870 eV/atom for the “11-dd” ribbon, closely approaching the cohesive energy of the α-boron sheet, whereas the “10-aa” ribbon yields 5.707 eV/atom. The higher stability of “dd” edges can be attributed to their enhanced structural uniformity and minimal edge reconstruction. The double chain termination effectively saturates most of the dangling bonds at the boundary, reducing electronic localization and strain, which leads to higher binding energy. Moreover, the smaller edge energy (ε ≈ 0.20 − 0.21 eV/Å) observed for “dd” ribbons confirms that “dd” edges are more favorable. In contrast, the armchair—armchair chain (“aa”) configuration has the lowest $E_{b}$ values (5.386–5.798 eV/atom), corresponding to the largest edge energy (ε ≈ 0.58 eV/Å). The armchair chain edges host a large number of under-coordinated atoms and irregular bond lengths ranging from 1.52 Å to 1.94 Å (Table 1), signifying enhanced local stress and substantial bond-length variation. This geometric diversity introduces strain energy and localized electronic states, both of which destabilize the structure. Therefore, the “aa” BNRs are the least stable among all edge terminations. Between these extremes, there are several intermediate edge configurations that bridge the stability gap. The single chain (“ss” and “as”) and double chain (“ad” and “ds”) ribbons show progressively increasing $E_{b}$ values. For instance, “ad” BNRs exhibit $E_{b}$ = 5.367 − 5.837 eV/atom with ε ≈ 0.41 − 0.45 eV/Å, while “ds” shows $E_{b}$ = 5.645 − 5.858 eV/atom and ε ≈ 0.33 − 0.35 eV/Å. The edge energies follow the trend as “dd” < “ds” < “ad” < “ss” < “as” ≈ “aa”. This systematic decrease in edge energy (ε) confirms the energetic hierarchy deduced from the binding energy ($E_{b}$) trends. The $E_{b}$ follows the order “aa” < “as” < “ss” < “ad” < “ds” < “dd”, which clearly shows an increasing stabilization as edge atoms become more coordinated. These trends correspond to a gradual reduction in the number of unsaturated valences and dangling bonds along the edge.

A complementary understanding arises from examining bond-length variation at the ribbon edges. Edge atoms in “aa” BNRs display the broadest bond-length range (1.52–1.94 Å), indicating significant structural distortions and local stress accumulation. But “dd” edges maintain a narrower and more uniform distribution (1.64–1.73 Å), suggesting nearly bulk-like coordination and reduced edge reconstruction. This structural homogeneity translates directly to improved binding energy, highlighting that uniform bonding environments at the edges are a key factor governing nanoribbon stability. The more regular the bond pattern, the lower the total energy, since localized distortions and under-coordination are minimized.

The “zz” BNRs obtained from zigzag directions show remarkably high binding energies ($E_{b}$ = 5.727 − 5.876 eV/atom), exceeded only by the “dd” ribbons. The corresponding edge energies (ε ≈ 0.34 eV/Å) make them one of the most stable terminations. This suggests that the zigzag-type edges allow efficient reconstruction and electron delocalization, which effectively saturate edge bonds. Overall, both energetic and structural analyses show a clear stability trend among all investigated BNRs. Based on edge energy (ε) and binding energy ($E_{b}$), the order of thermodynamic favorability follows: “dd” (most stable) < “zz” < “ds”< “ad” < “ss” < “as” < “aa” (least stable).

Since passivation with different atoms can enhance the stability of pristine BNRs by saturating dangling bonds and lowering edge energy, we further investigated the role of edge passivation on the stability of the “zz” BNRs with hydrogen (H), Oxygen (O), and Fluorine (F) at the edges. In Table 2, the formation energy of passivation per unit length is presented. The formation energy of passivation per unit length is calculated using the formula $E_{f}=(E_{BNR+X}\;-n_{X}\times E_{X}\;-\;E_{BNR})/L$; $E_{BNR+X}$ energy of passivated BNRs, $E_{BNR}$ is the energy of pristine BNRs, $n_{X}$ is no of passivated X (X = H-, O-, F-) atom, $E_{X}$ is the energy of an isolated X atom, and L is the length of the unit cell. As shown in Table 2, all the passivated “X-N-zz” BNRs exhibit negative formation energies, which confirms that H-, O-, and F- passivation processes are energetically favorable. Among them, fluorine-passivated BNRs show the highest negative formation energy (E_f_ = −1.415 to −1.429 eV/Å), indicating the strongest stabilization, followed by oxygen and hydrogen passivations. The formation energy E_f_ oscillates with ribbon width. This peculiar behavior indicates a stronger electronic correlation between the edge passivated atoms and the inner boron atoms in the “zz” BNRs.

**Figure 3:**
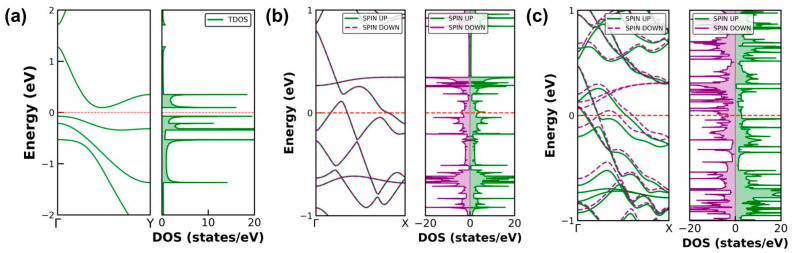
(**a**) Electronic band structure and total density of states of the “3-ad” nanoribbon, showing a finite band gap. (**b**,**c**) Spin-resolved band structure and DOS of the “8-zz” and “17-zz” nanoribbons, respectively, symmetric and asymmetric DOS show AFM and FM behavior of the BNRs.

The calculated electronic band structure and density of states (DOS) for all the BNRs are shown in Figure 3 (and in Supplementary Figure S1). The results reveal that most of the BNRs are metallic, with multiple bands crossing the Fermi level. Only “3-ad” BNR has an indirect band gap of 0.165 eV. This gap closes with increasing BNR width. There is no evidence of Peierls-type distortions. Even after edge passivation with hydrogen (H), oxygen (O), and fluorine (F) atoms, all BNRs retain their metallic character. This indicates band structure is robust against passivation, highlighting their potential for nanoscale electronic and spintronic applications.

**Figure 4:**
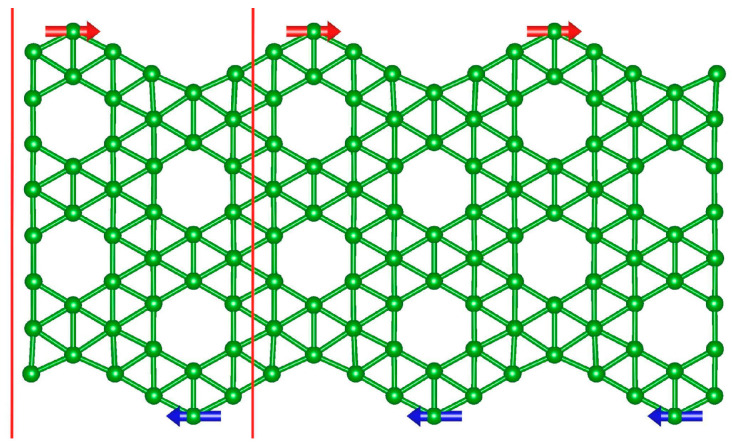
Magnetic ordering in “8-zz” BNRs. The red and blue arrow shows antiferromagnetic spin alignment at the opposite edges. The red line shows the unit cell.

Magnetic moments indicate that ribbons cut along the armchair(ac) direction are predominantly non-magnetic or only weakly magnetic. In contrast, zigzag-edge boron nanoribbons (“zz”-BNRs) display a pronounced width-dependent anisotropy. As summarized in Table 1, the narrow “5-zz” ribbon is non-magnetic, consistent with earlier reports [21]. With increasing width, “zz” BNRs develop magnetic ground states: “8-zz”, “11-zz”, and “14-zz” show ferromagnetic alignment along each edge with antiferromagnetic coupling between opposite edges (i.e., AFM across the ribbon, as shown in Figure 4), whereas “17-zz” is ferromagnetic both along and across the edges. The total magnetic moment per atom is largest for “8-zz” (1.335 $\mu_{B}/atom$) and decreases slightly with width, reaching 1.30 $\mu_{B}/atom$ for “17-zz”. Edge passivation of hydrogen (H) and fluorine (F) suppresses the magnetism on edges, and “zz” BNRs become non-magnetic. In contrast, passivation of oxygen (O) at the edges retains finite magnetism. Pristine “5-zz” is non-magnetic, but it becomes magnetic with O-passivation (“O-5-zz”) with a magnetic moment are 0.855 $\mu_{B}/atom.$ The magnetic moment for other O-passivated “zz” are shown in Table 2 and magnetic alignments are the same as pristine “zz” BNRs.

To quantify the inter-edge exchange, we evaluate $\Delta {E_{b}}^{AFM-FM}=\;{E_{b}}^{AFM}-{E_{b}}^{FM}$. The energy differences are 0.8 meV (“8-zz”), 0.172 meV (“11-zz”), 0.015 meV (“14-zz”) and −0.02 meV (“17-zz”). The progressive reduction of $\Delta {E_{b}}^{AFM-FM}$ signifies a gradual weakening of AFM inter-edge coupling with greater width, and a crossover to a slightly FM-favored state at “17-zz”. For the passivated “zz” BNRs, the corresponding energy differences are 0.3 meV (“O-5-zz”), 3.2 meV (“O-8-zz”), −0.01 meV (“O-11-zz”), 0.005 meV (“O-14-zz”) and 0.003 meV (“O-17-zz”). These results confirm that all O-passivated BNRs except “O-11-zz” favor AFM inter-edge coupling.

These findings highlight the tunability of both electronic and magnetic properties in boron nanoribbons by controlling their width and edge geometry. The emergence of magnetism in zigzag (zz) nanoribbons with decreasing width, along with the ability to transition between AFM and FM orderings, points to their potential utility in nanoscale spintronic devices.

The electronic transport properties of α-BNRs were studied in the ballistic regime since mean free paths in similar BNRs are predicted to be on the order of ~30–100+ nm [20,38], exceeding the nanowire dimension. Under the ballistic transport assumption, the ribbon’s conductance is determined by the number of conducting channels at or near E_F_. For a single conducting channel, the conductance is $G_{0}=\frac{{2e}^{2}}{h}=7.748\times 10^{-5}\;S$, accounting for spin degeneracy. With the exception of the semiconducting “3-ad”, all nanoribbons have at least three spin-degenerate channels at E = E_F_, as shown in Table 1, which can support ~1 µA under a small bias of 4 mV. The “zz” BNRs show different numbers of open channels depending on spin. Due to the high conductivity of these nanoribbons, they can operate as low-contact resistance [39] interconnects for 2D electronics with very little power loss.

Since bulk borophene is metallic, the number of transmission channels is expected to scale linearly with width. However, as shown in Table 1, the BNRs in this study do not show any strong width dependence of T(E = E_F_), indicating the dominance of confinement or edge effects on the transmission of low-energy carriers in α-BNRs. As the nanoribbons increase in width, we expect the transmission to return to a linear dependence on width, independent of edge structure. At energies away from E_F_, the transmission depends strongly on the width.

**Figure 5:**
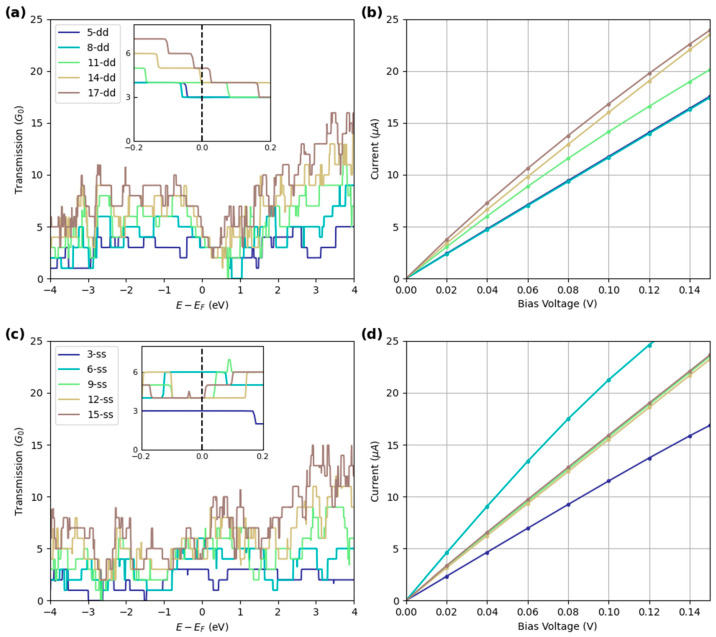
(**a**) Transmission at V = 0 for various “dd” BNRs. Inset shows transmission close to E_F_. Within the “dd” edge nanoribbons structures, T(E = E_F_) slightly increases with width. (**b**) I-V characteristics of “dd” edge nanoribbons. (**c**) Transmission at V = 0 for various “ss” BNRs. slightly increases with width. (**d**) I-V characteristics of “ss” edge nanoribbons.

**Figure 6:**
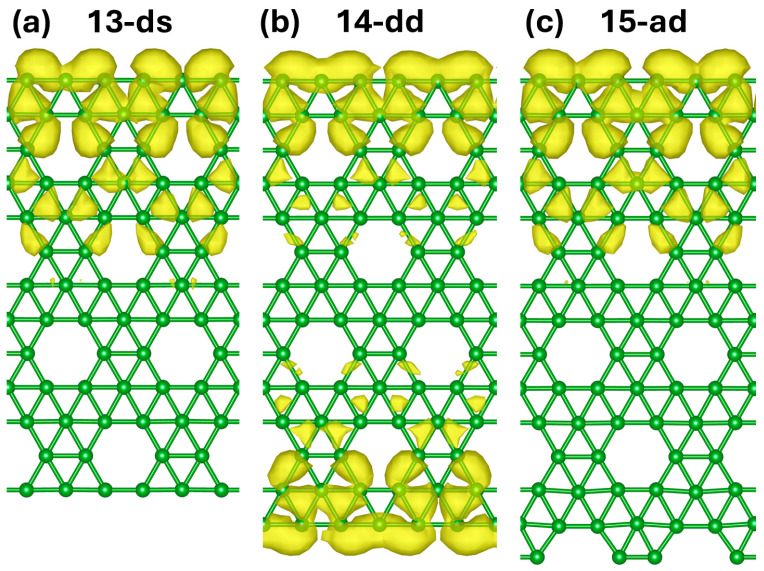
Magnitude of the “d” edge state wavefunction on (**a**) “13-ds”, (**b**) “14-dd”, and (**c**) “15-ad” nanoribbons.

The transmission plots for “dd” and “ss” BNRs of increasing widths are shown in Figure 5. In the “dd” edge nanoribbons, a weak dependence on width at E_F_ is shown. However, in the “ss” BNRs, the second-smallest (“6-ss” BNR) has the highest transmission near E_F_. Notably, the “6-ss” BNR has double the transmission at E_F_ compared to ribbons almost 3× its width. Careful selectivity of ribbon width and edge construction may be necessary for optimized and consistent BNRs for electronic devices. It is apparent from Figure 5a and Figure 6c that the transmission away from E_F_ is more dependent on width, owing to more total electrons, and thus channels, in the wider ribbons. Transmission plots for other edge configurations are shown in Supplementary Figures S2–S5.

The spatial distribution of transmission channels was analyzed using Inelastica [36,37]. Several edge-localized transmission channels at E = E_F_ were identified and are shown in Figure 6 and Figure S6. The “s” and “d” edge channels persist independently of the structure of the opposite edge. The “a” edge shows transmission channel wavefunctions with higher magnitude on the edges, but not fully localized. These edge channel observations agree with previous studies suggesting electron density build-up at other BNRs edges [16]. In the thinnest ribbons, it is difficult to separate the edge orbitals from the non-edge orbitals from differences in magnitude of the wavefunctions. However, the stability of transmission at widths even under 0.5 nm indicates promising applications of these BNRs as ultrathin 1D wires.

Note that these edge states are a result of hybridized orbitals of under-coordinated edge atoms and are by no means topologically protected. This is to say that disorder or substrate interactions can disrupt these edge channels. Additionally, many of the transmission channels, especially in the thinner nanoribbons, correspond to wavevectors in the middle of the Brillouin zone, with real space wavelengths of a few nanometers. In such cases, the coherence across the channel may easily be disrupted by finite length nanoribbons and imperfect coupling across junctions. Careful mode-matching at interfaces may be necessary to fully utilize the conducting channels of BNRs.

Spin-polarized transport calculations were performed for the “zz” BNRs structures. Due to the magnetization on the edges, the transmission curves show clear spin dependence. Interestingly, the minority spin carrier (spin down) has increased transmission at E_F_. These magnetic nanoribbons show promise for use as a spin filter with polarization magnitude up to 42.8% at E = E_F_, quantified as follows:

(2) $$P(E)=\frac{T_{↑}(E)-T_{↓}(E)}{T_{↑}(E)+T_{↓}(E)}$$

where $T_{↑}(E)$ and $T_{↓}(E)$ are the majority (spin up) and minority (spin down) transmissions.

**Figure 7:**
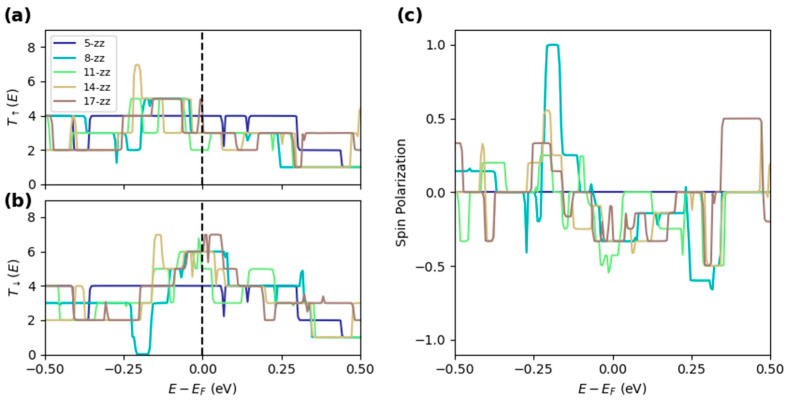
Transmission function for (**a**) spin up and (**b**) spin down channels for “zz” BNRs of increasing width. (**c**) Spin polarization, per Equation (2), as a function of energy.

Figure 7 below shows the spin-resolved transmission across “zz” BNRs widths. Again, there is no strong dependence of width on the total transmission at E_F_. The spin-dependent transport in magnetic BNRs has shown promise in spintronics applications [38,40]. Although the spin polarization is moderate in the intrinsic nanoribbons, external modulations, including gate control, doping, and phase junctions, offer promising methods to tune the electronic and spin transport properties [20,40,41]. Looking at the spin polarization of the thinnest magnetic ribbon, “8-zz”, we observe a swing from 100% to −60% polarization within +/−0.25 eV of the intrinsic Fermi level. The wider nanoribbons also show a similar flip of the polarization, but no half-metallic region characterized by +/−100% polarization. This suggests that spin filtering effects can be dynamically modulated or flipped using finite bias or gating, opening up applications for BNRs spintronic devices.

Upon passivation of the “zz” BNRs with hydrogen or fluorine, the ribbons become non-magnetic and thus lose spin transport properties, as shown in Table 2. Additionally, the transmission of the wider is suppressed to only two spin-degenerate channels near the Fermi level, which is less conductive than all of the unpassivated BNR structures except the insulating “3-ad” BNR. On the other hand, the “O-zz” BNR structures are all magnetic.

## 4. Summary and Conclusions

In this work, we conducted a thorough investigation of α-borophene-based nanoribbons. We revealed how their structural, electronic, magnetic, and transport properties depend on edge shape and ribbon width. Our detailed analysis across seven edge families shows a clear energetic trend: “dd” > “zz” > “ds” > “ad” > “ss” > “as” > “aa”. This trend corresponds to lower edge stress and better coordination. The double chain “dd” edges have the highest binding energies (up to 5.90 eV/atom) and the lowest edge energies (≈0.20 eV Å^−1^). This confirms their strong thermodynamic stability and consistent structural uniformity. The steady increase in binding energy with ribbon width suggests that wide α-BNRs can be made with good stability, especially under conditions that promote self-saturation of dangling bonds. Electronic structure calculations show that metallic behavior is stable across all edge types. Even after hydrogen, oxygen, or fluorine passivation, the α-BNRs still have delocalized conduction channels at the Fermi level. The narrowest “3-ad” ribbon does open a small 0.165 eV gap, but this quickly disappears as the width increases. This enduring metallicity, along with edge-localized conduction states, explains the low resistance of α-BNRs and provides a simple way to understand their adjustable conductance. Magnetic analyses reveal width-dependent magnetism in zz nanoribbons. Narrow “zz” BNRs do not show magnetism, but as the width increases, localized edge moments appear. These moments shift from antiferromagnetic coupling between opposite edges to ferromagnetic alignment at both edges. The magnetic moment per atom (≈1.3 μ_B_) and the small differences between AFM and FM energy (less than 1 meV) suggest that the magnetic order can be easily switched using external fields or strain. Edge passivation has a strong impact on magnetism. Hydrogen and fluorine atoms reduce local moments, while oxygen termination secures finite magnetic states, allowing for chemical control of spin order. Ballistic transport calculations show that most α-BNRs have at least three conduction channels at E = E_F_, leading to conductance values of G ≈ 2–3 G_0_ (G_0_ = 2e^2^/h). The transmission spectra show little change with width near *E*_F_ but show significant variation at higher energies, indicating that both edge and bulk-like channels exist. Spin-resolved transport for magnetic “zz” BNRs shows polarization above 40%, confirming their spin filtering ability and suggesting potential applications as spin valves or thermospintronic devices.

Overall, these findings position α-borophene nanoribbons as an appealing option for one-dimensional metallic interconnects and spintronic components. Their strong intrinsic stability, controllable edge magnetism, and strong metallicity combine the benefits of graphene nanoribbons with the chemical flexibility of boron. Future research should examine how substrates, strain engineering, and heterostructure design influence their transport and magnetic properties. Creating α-BNRs with controlled edge chemistry could lead to advanced nanoscale conductors, spin filters, and quantum devices built on light, flexible boron structures.

## Figures and Tables

**Table 1:** Geometric, energetic, magnetic, and electronic transport properties of *α*-borophene nanoribbons (BNRs) with various edge configurations and widths. For each structure, we report the ribbon width (in Å), binding energy per atom ($E_{b}$, in eV/atom), edge energy (ε, in eV/Å), minimum and maximum bond lengths (d_min_ and d_max_, respectively), total magnetic moment (M, in *μ*_B_), and the number of transmission channels T at the Fermi level (E = E_F_), (up/down) is transmission channels of up spin and down spin of “zz” BNRs. Structures are grouped by edge type: “aa”, “as”, “ad”, “ss”, “ds”, “dd”, and “zz”, as defined in the text.

Name	Width (Å)	$E_{b}\;$(eV/atom)	ε (eV/Å)	d_min_ (Å)	d_max_ (Å)	M ($\mu_{B}/atom$)	T (E = E_F_) (up/down)
4-aa	4.11	5.386	0.52	1.54	1.94	0.012	3
7-aa	8.56	5.599	0.59	1.53	1.79	0.028	3
10-aa	12.96	5.707	0.59	1.52	1.80	0.017	3
13-aa	17.34	5.764	0.58	1.52	1.80	0.003	3
16-aa	21.73	5.798	0.58	1.52	1.80	0.001	3
5-as	5.79	5.507	0.56	1.51	1.79	0.019	4
8-as	10.18	5.674	0.55	1.51	1.79	0.006	3
11-as	14.55	5.749	0.55	1.51	1.79	0.004	3
14-as	18.94	5.791	0.55	1.51	1.79	0.000	4
3-ad	2.94	5.367	0.45	1.54	1.78	0.000	0
6-ad	7.28	5.669	0.43	1.52	1.79	0.000	4
9-ad	11.67	5.765	0.42	1.52	1.80	0.000	4
12-ad	16.06	5.811	0.41	1.52	1.79	0.000	4
15-ad	20.45	5.837	0.41	1.52	1.79	0.003	5
3-ss	2.98	5.379	0.46	1.60	1.70	0.015	3
6-ss	7.41	5.630	0.50	1.60	1.72	0.000	6
9-ss	11.77	5.733	0.50	1.61	1.71	0.000	4
12-ss	16.17	5.785	0.50	1.62	1.71	0.000	4
15-ss	20.53	5.816	0.49	1.62	1.74	0.000	4
4-ds	4.45	5.645	0.33	1.61	1.75	0.011	3
7-ds	8.87	5.758	0.35	1.62	1.74	0.019	4
10-ds	13.27	5.810	0.35	1.61	1.73	0.004	5
13-ds	17.65	5.839	0.35	1.62	1.73	0.003	5
16-ds	22.05	5.858	0.35	1.62	1.74	0.002	4
5-dd	5.98	5.800	0.20	1.64	1.72	0.009	3
8-dd	10.38	5.844	0.21	1.64	1.72	0.005	3
11-dd	14.78	5.870	0.21	1.65	1.73	0.000	4
14-dd	19.13	5.886	0.21	1.65	1.72	0.003	4
17-dd	23.52	5.895	0.21	1.66	1.72	0.002	5
5-zz	9.03	5.727	0.34	1.60	1.86	0.005	4
8-zz	14.13	5.802	0.35	1.62	1.83	1.335	3/6
11-zz	19.18	5.840	0.34	1.62	1.82	1.335	2/5
14-zz	24.24	5.862	0.34	1.62	1.82	1.325	3/6
17-zz	29.29	5.876	0.34	1.62	1.81	1.30	3/6

**Table 2:** For passivated “zz” structure, we report the formation energy per unit length (E_f_, in eV/Å), bond lengths between B and X atom (X = H-, O-, F-), total magnetic moment (M, in μ_B_), and the number of transmission channels T at the Fermi level (E = E_F_), (up/down) is transmission channels of up spin and down spin of passivated BNRs.

	E_f_ (eV/Å)	d_B-X_ (Å)	M ($\mu_{B}/atom$)	T (E = E_F_) (up/down)
H-5-zz	−0.916	1.189	0.0	3
H-8-zz	−0.931	1.189	0.0	5
H-11-zz	−0.935	1.189	0.0	2
H-14-zz	−0.936	1.189	0.0	2
H-17-zz	−0.937	1.189	0.0	2
O-5-zz	−1.342	1.283	0.855	3/5
O-8-zz	−1.338	1.283	0.791	4/6
O-11-zz	−1.349	1.283	0.845	6/4
O-14-zz	−1.347	1.283	0.855	2/6
O-17-zz	−1.345	1.283	0.87	2/6
F-5-zz	−1.415	1.339	0.0	3
F-8-zz	−1.428	1.339	0.0	4
F-11-zz	−1.429	1.339	0.0	2
F-14-zz	−1.428	1.339	0.0	2
F-17-zz	−1.428	1.339	0.0	2

## Data Availability

The original contributions presented in this study are included in the article. Further inquiries can be directed to the authors.

## Supplementary Materials

The following supporting information can be downloaded at the following: https://www.mdpi.com/article/10.3390/molecules30214177/s1, Figure S1: (a) Electronic band structure and density of states (DOS) of “aa” BNRs, (b) Electronic band structure and density of states (DOS) of “as” BNRs, (c) Electronic band structure and density of states (DOS) of “ad” BNRs, (d) Electronic band structure and density of states (DOS) of “ss” BNRs, (e) Electronic band structure and density of states (DOS) of “ds” BNRs, (f) Electronic band structure and density of states (DOS) of “dd” BNRs, (g) Electronic band structure and density of states (DOS) of “zz” BNRs, (h) Electronic band structure and density of states (DOS) of H-passivated “zz” BNRs, (i) Electronic band structure and density of states (DOS) of O-passivated “zz” BNRs, (j) Electronic band structure and density of states (DOS) of F-passivated “zz” BNRs; Figure S2: (a) Transmission at V = 0 for various “aa” edge nanoribbons. Inset shows no width dependence for transmission near E_F_; T(E = E_F_) = 3 for all 5 “aa” edge nanoribbons. (b) I-V characteristics of aa-BNRs. “7-aa” and “10-aa” show non-linear I-V due to the existence of band edges near E_F_. The equilibrium T(E) of “10-aa” shows a spike near E_F_ due to a flat region in the band structure near E_F_, but it does not play any role in the transport at finite voltages; Figure S3: (a) Transmission at V = 0 for various “as” edge nanoribbons. Inset shows transmission near E_F_. (b) I-V characteristics of “as” edge nanoribbons; Figure S4: (a) Transmission at V = 0 for various “ad” edge nanoribbons. Inset shows transmission near E_F_. (b) I-V characteristics of “ad” edge nanoribbons; Figure S5: (a) Transmission at V = 0 for various “ds” edge nanoribbons. Inset shows transmission near E_F_. (b) I-V characteristics of “ds” edge nanoribbons; Figure S6: Edge-localized transmission channels for “ss” edge nanoribbons shown as absolute (left) and real (right) parts of the wavefunction. Transmission channels (b) and (c) have identical absolute wavefunctions, but their phases are symmetric/anti-symmetric across the ribbon width. When changing one edge, only one of (b) and (c) exists.
