# Supplementary material for: α-Borophene Nanoribbons: Edge-Dependent Metallic and Magnetic Properties for Low-Dimensional Nanoelectronics
Source: Molecules. 2025 Oct 24;30(21):4177. doi: 10.3390/molecules30214177 (PMC12610275; doi:10.3390/molecules30214177)
Supplement: Supplementary file 1 [file molecules-30-04177-s001.zip › molecules-3905136-supplementary.pdf]

α-Borophene Nanoribbons: Edge-Dependent Metallic and Magnetic Properties for Low-Dimensional Nanoelectronics

Subrata Rakshit [1,2], Favian Sun [2], Nevill Gonzalez Szwacki[1] and Boris I Yakobson[2,*]

[1]Faculty of Physics, University of Warsaw, Pasteura 5, PL-02093 Warszawa, Poland; gonz@fuw.edu.pl

[2] Department of Materials Science and NanoEngineering, Rice University, Houston, Texas 77005, USA

*Correspondence: biy@rice.edu

(a)

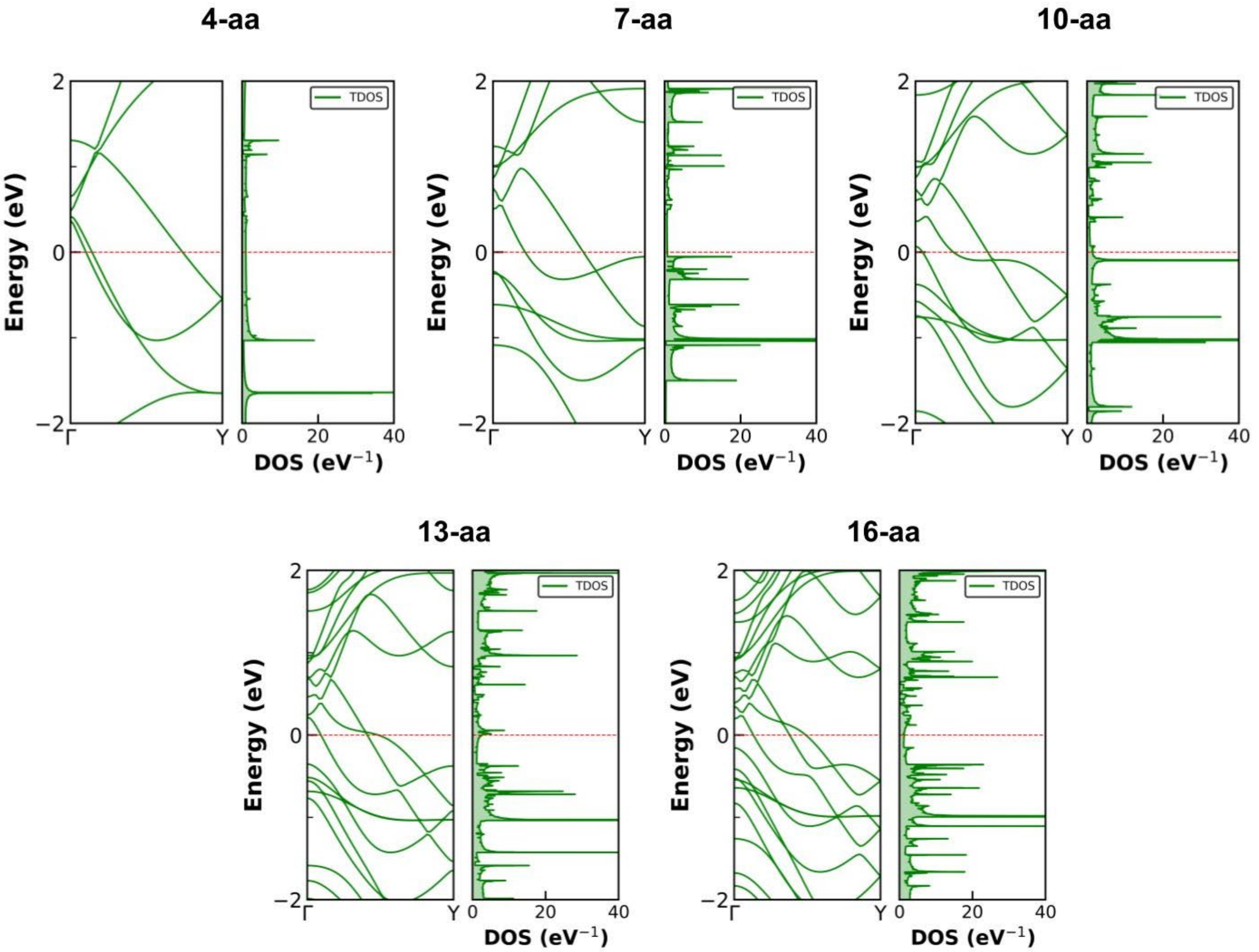

Fig S1(a): Electronic band structure and density of states (DOS) of “aa” BNRs.

(b)

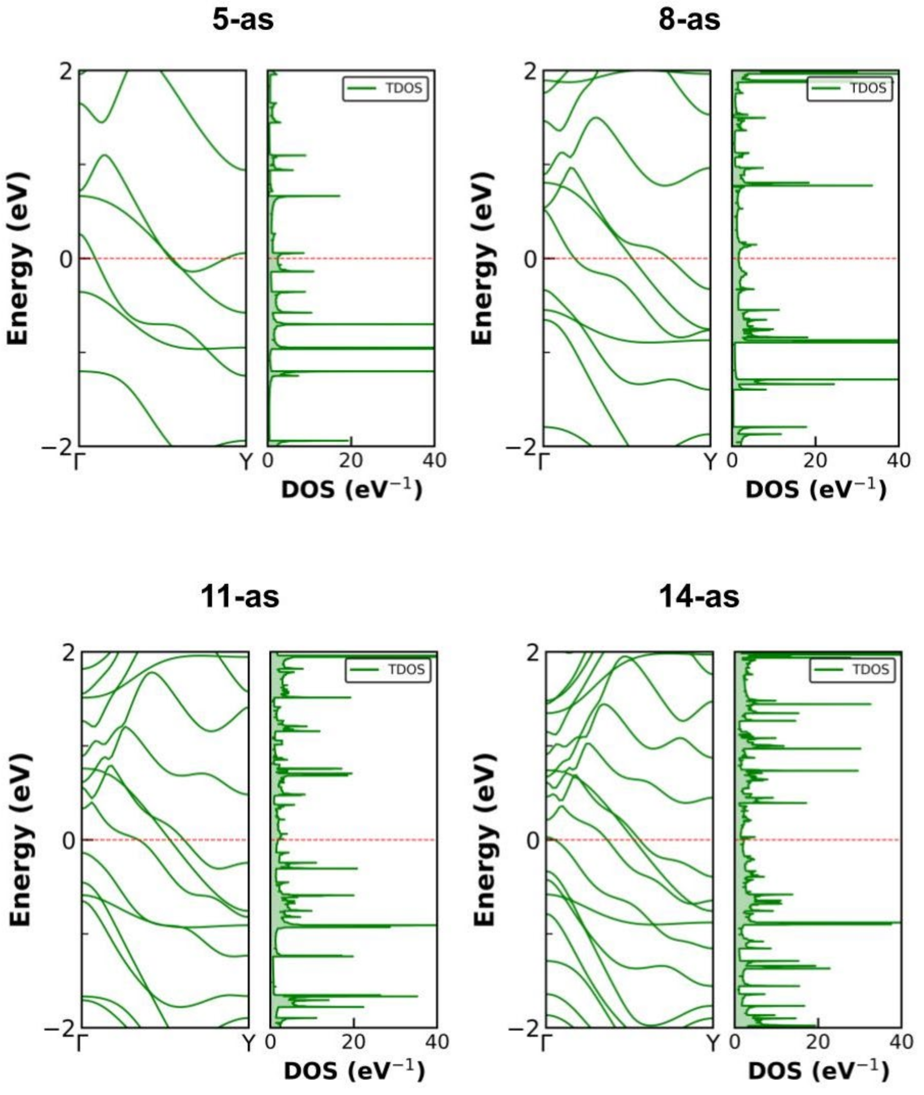

Fig S1(b): Electronic band structure and density of states (DOS) of “as” BNRs.

(c)

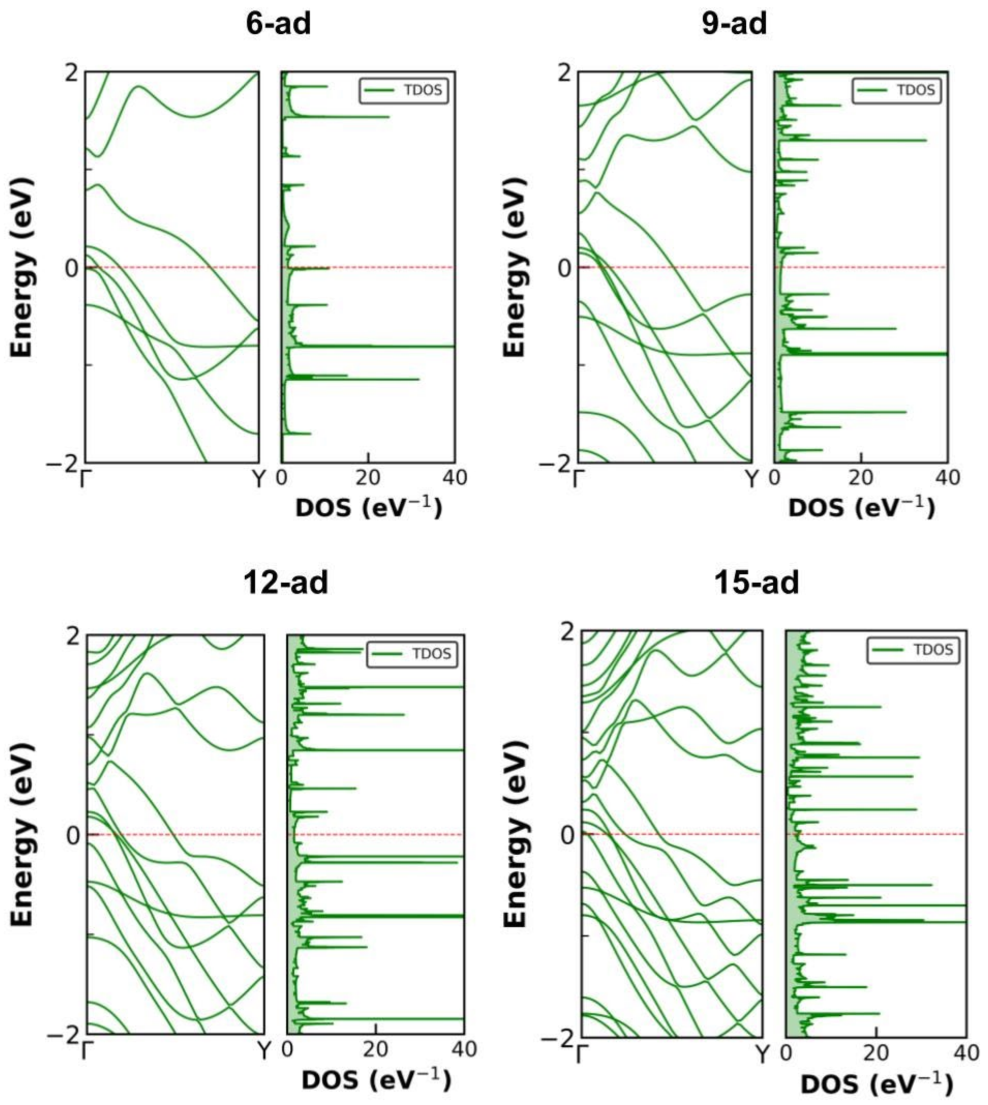

Fig S1(c): Electronic band structure and density of states (DOS) of “ad” BNRs.

(d)

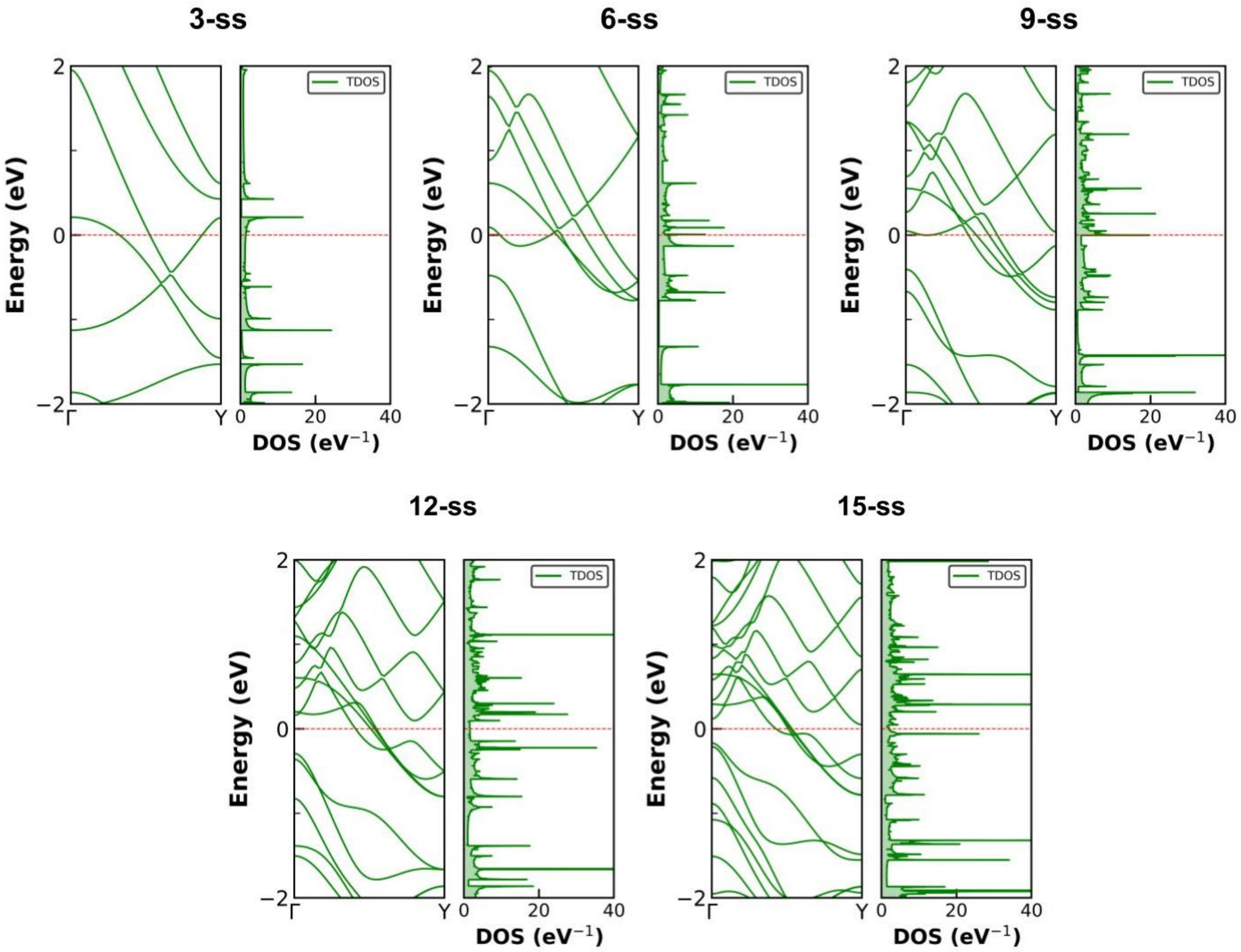

Fig S1(d): Electronic band structures and density of states (DOS) of “ss” BNRs.

(e)

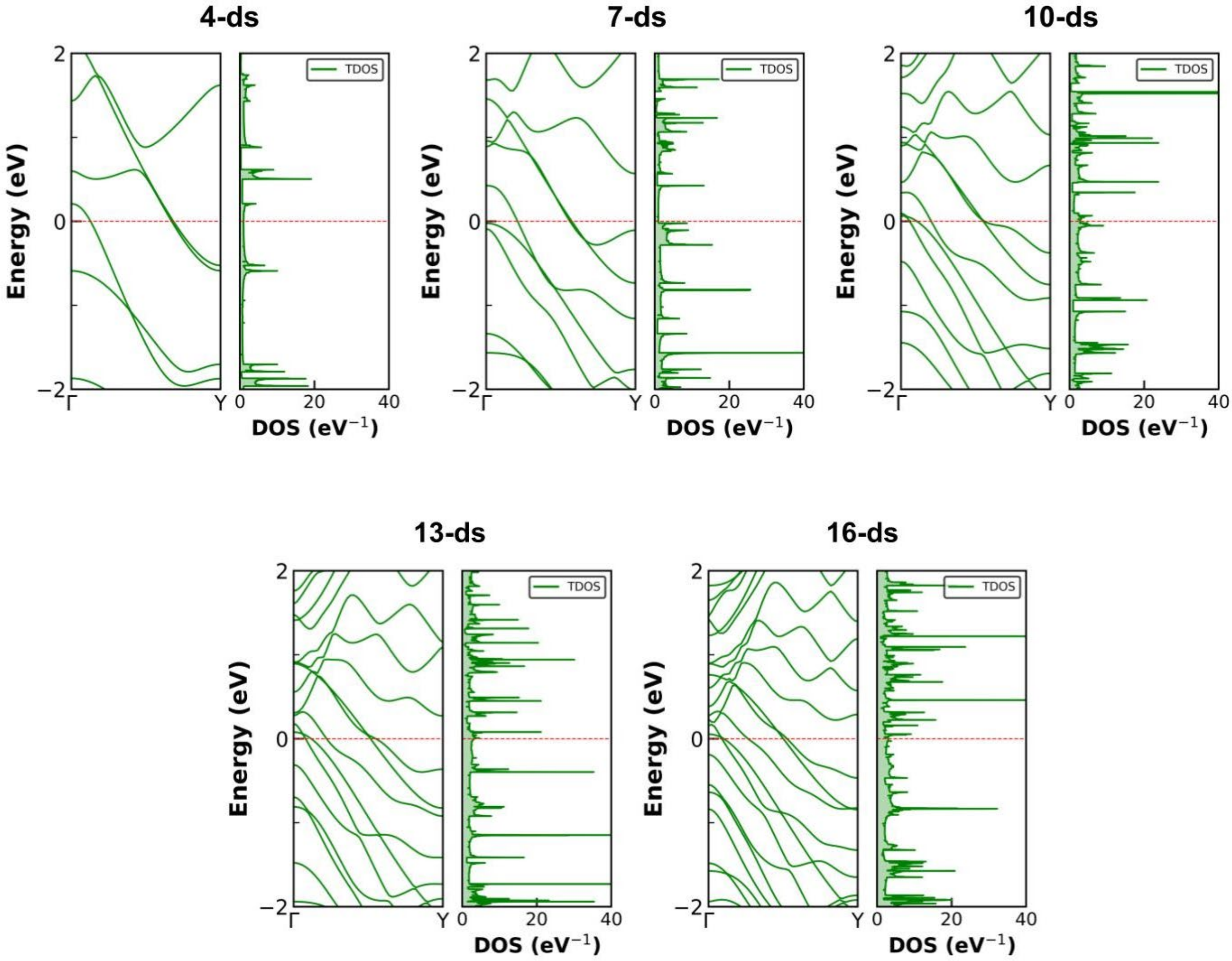

Fig S1(e): Electronic band structures and density of states (DOS) of “ds” BNRs.

(f)

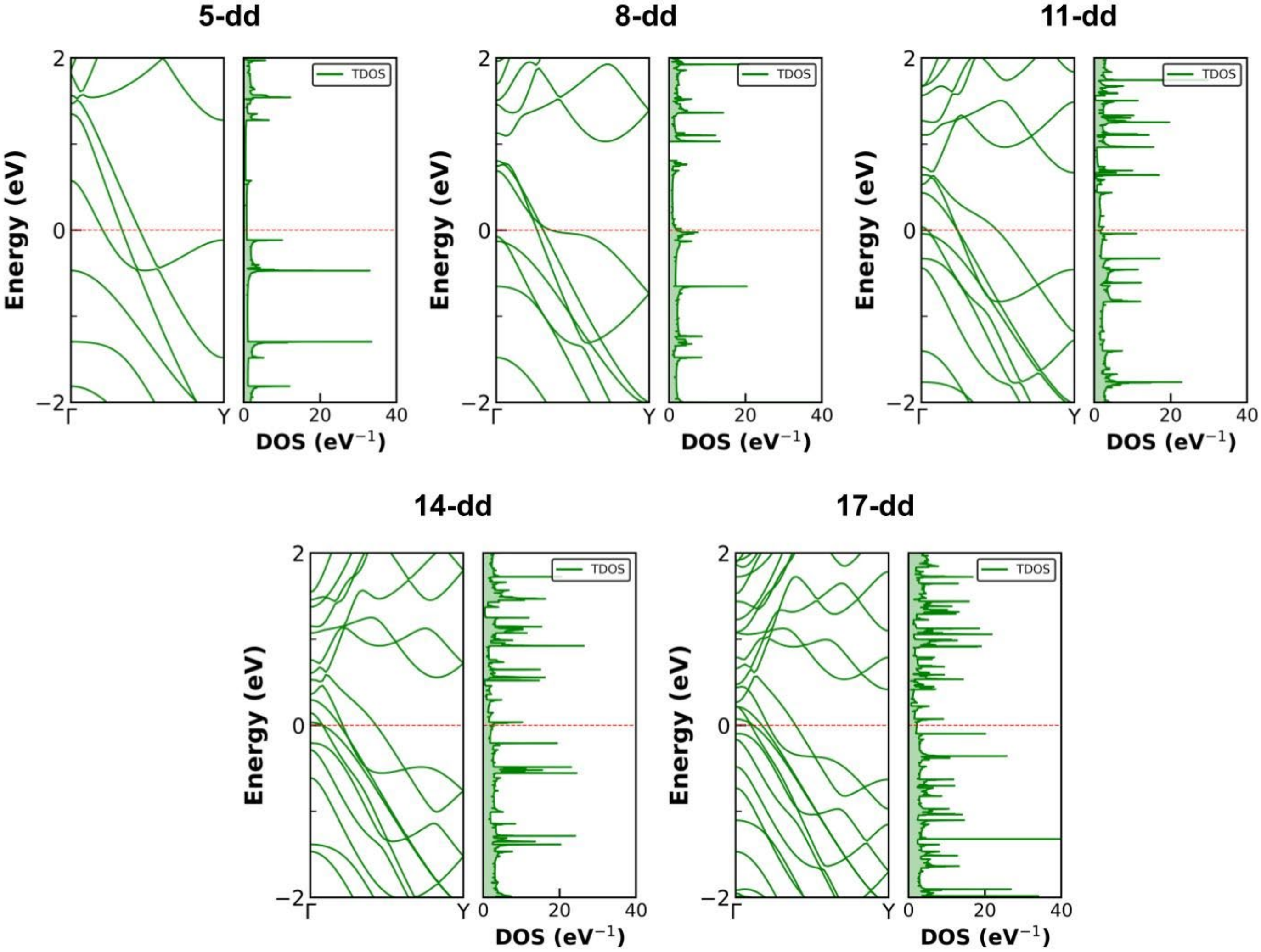

Fig S1(f): Electronic band structures and density of states (DOS) of “dd” BNRs.

(g)

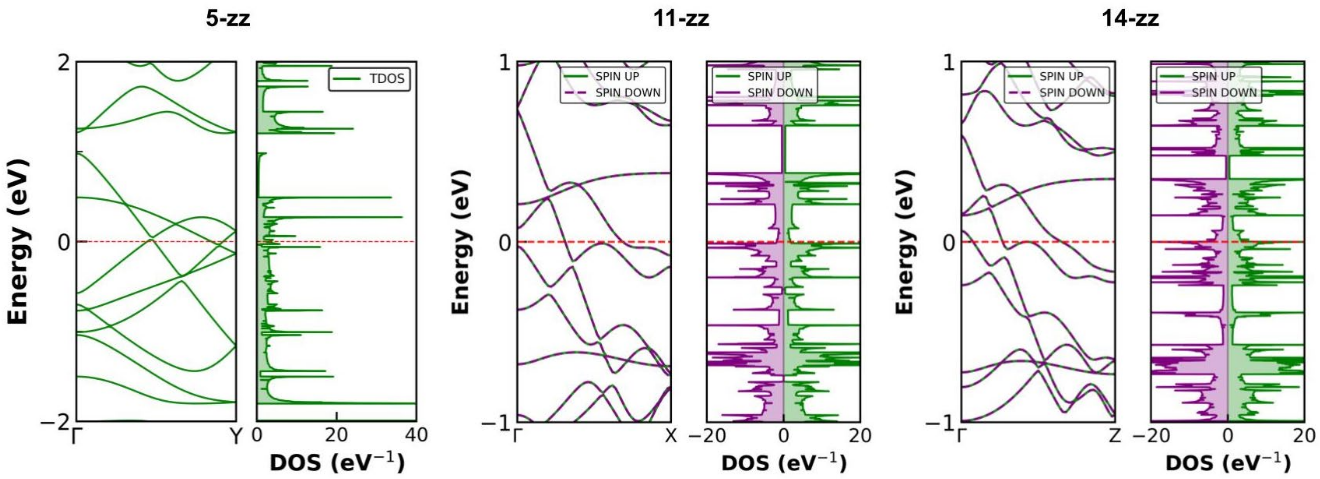

Fig S1(g): Electronic band structures and density of states (DOS) of “zz” BNRs.

(h)

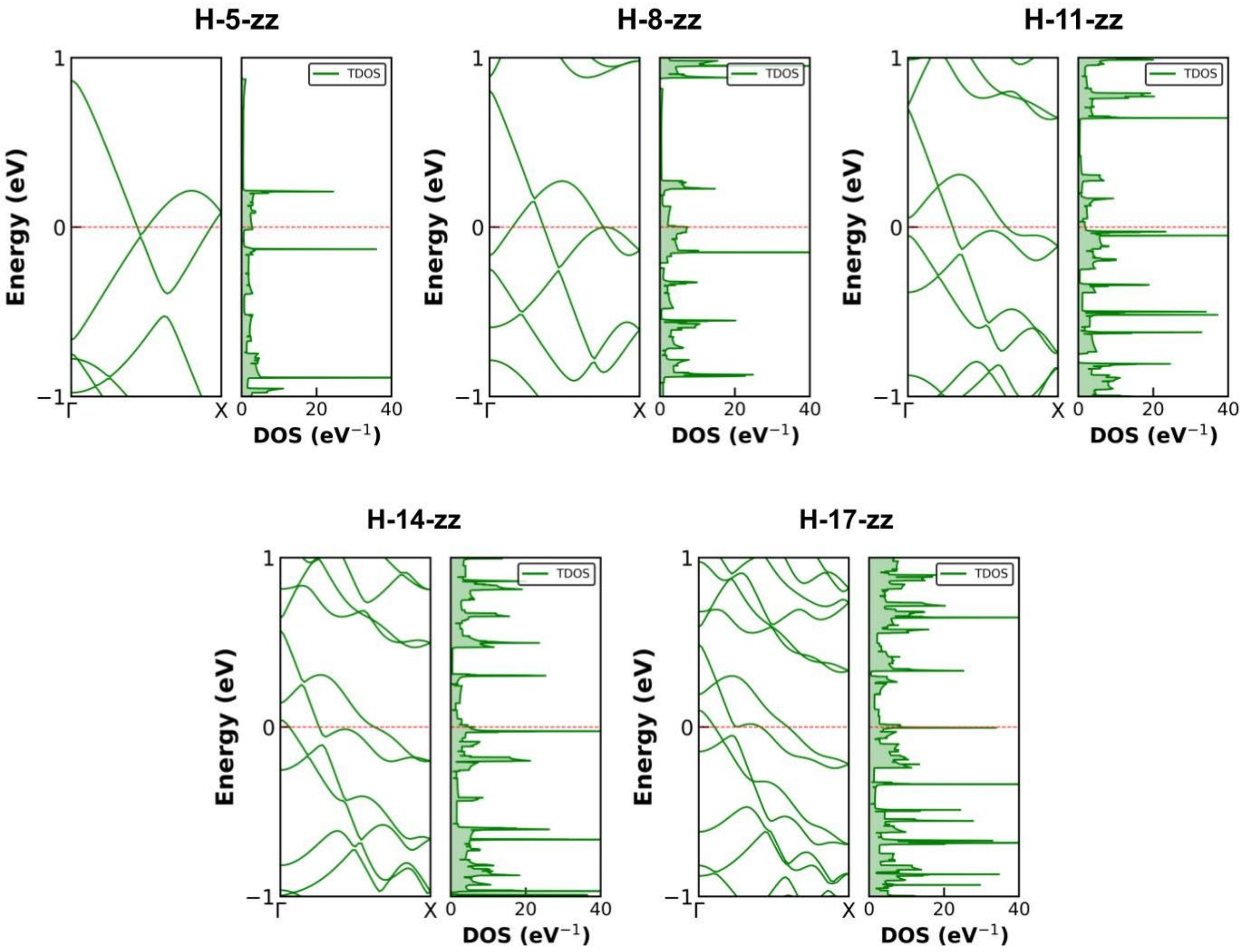

Fig S1(h): Electronic band structures and density of states (DOS) of H-passivated “zz” BNRs.

(i)

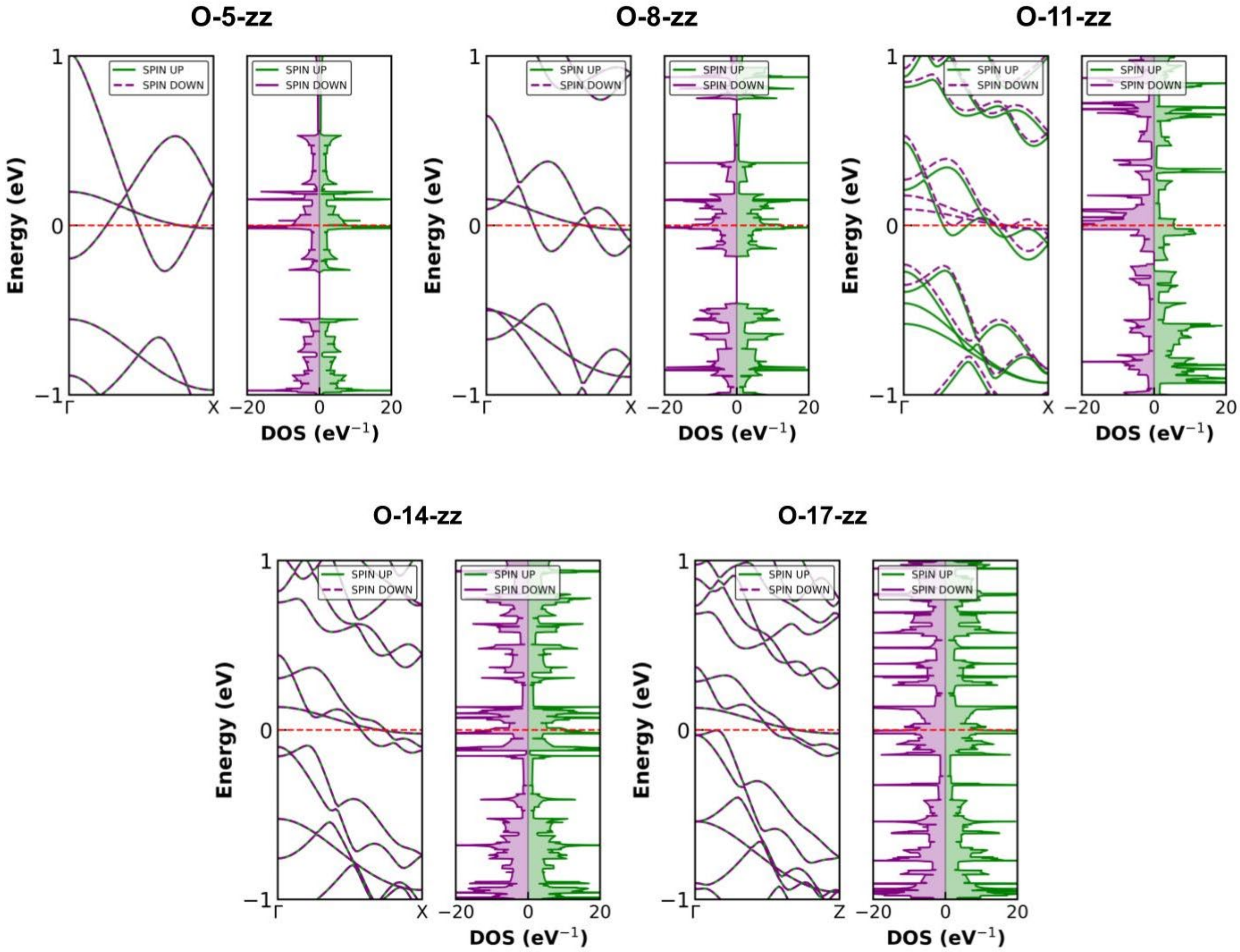

Fig S1(i): Electronic band structures and density of states (DOS) of O-passivated “zz” BNRs.

(j)

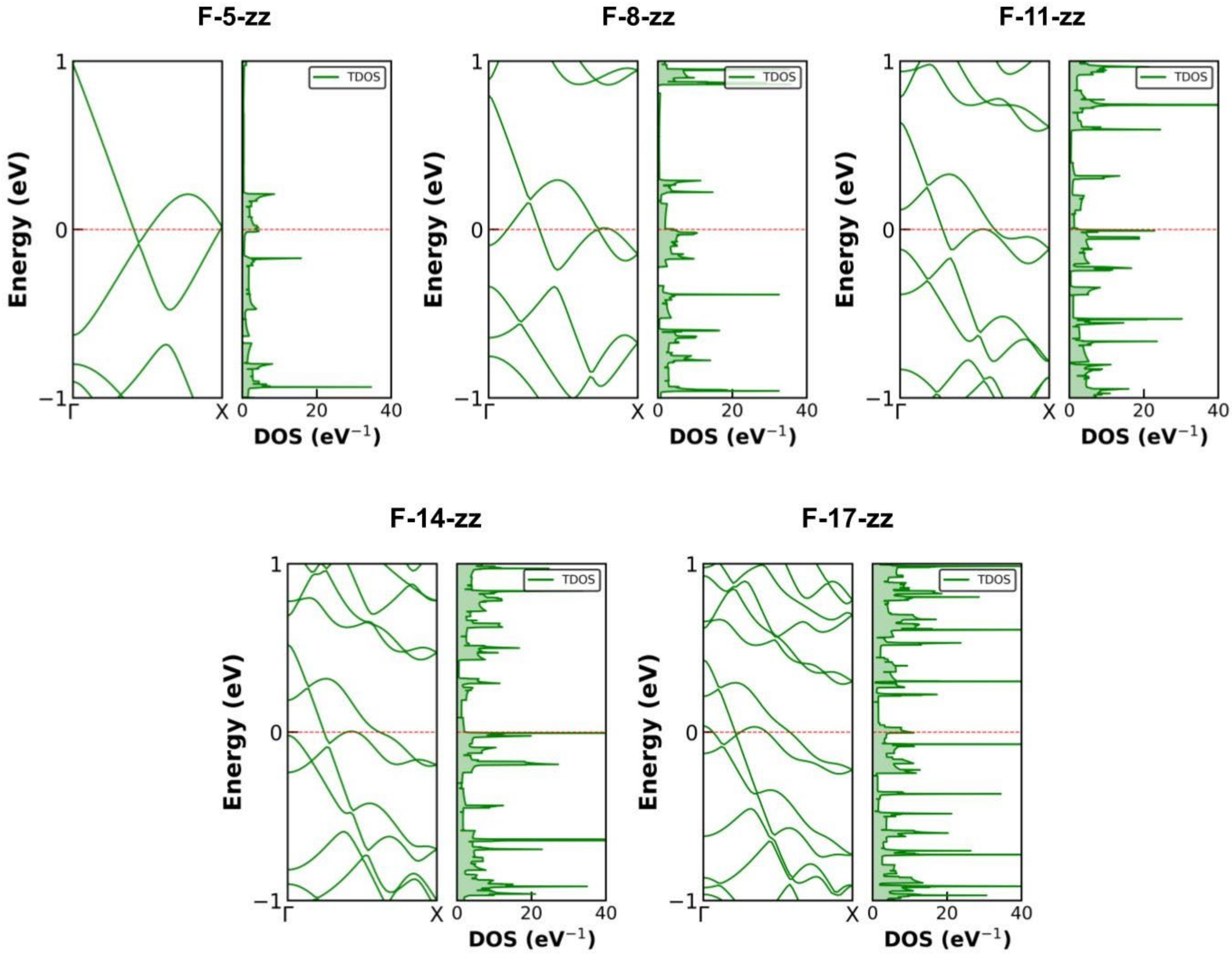

Fig S1(j): Electronic band structures and density of states (DOS) of F-passivated "zz" BNRs.

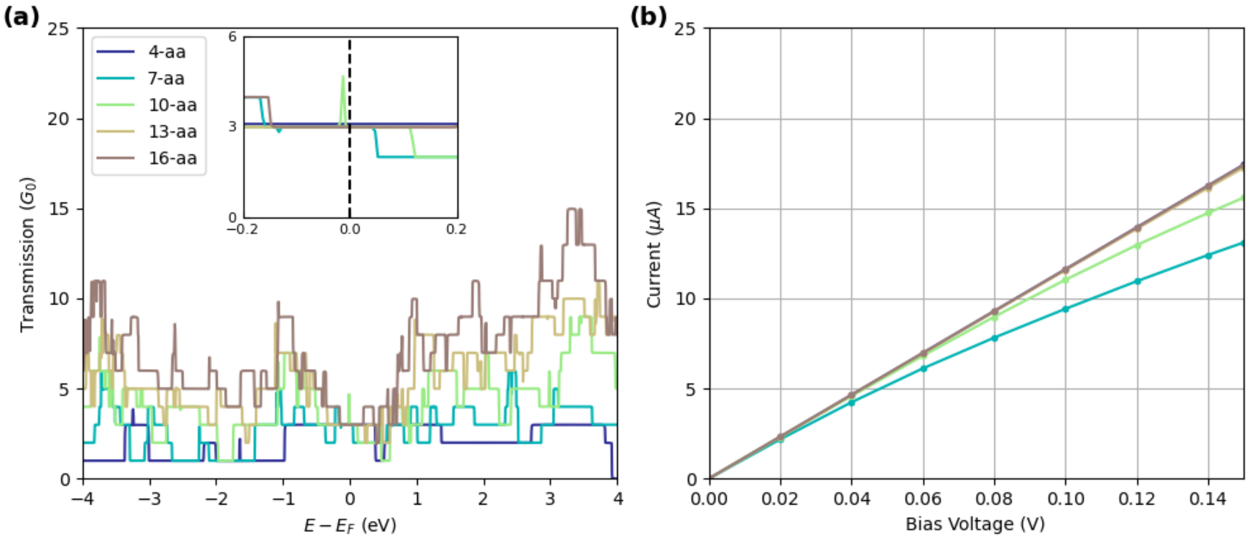

Fig S2: (a) Transmission at V=0 for various “aa” edge nanoribbons. Inset shows no width dependence for transmission near E_F; $T(E=E_F)=3$ for all 5 “aa” edge nanoribbons. (b) I-V characteristics of aa-BNRs. “7-aa” and “10-aa” show non-linear I-V due to the existence of band edges near E_F. The equilibrium T(E) of “10-aa” shows a spike near E_F due to a flat region in the band structure near E_F, but it does not play any role in the transport at finite voltages.

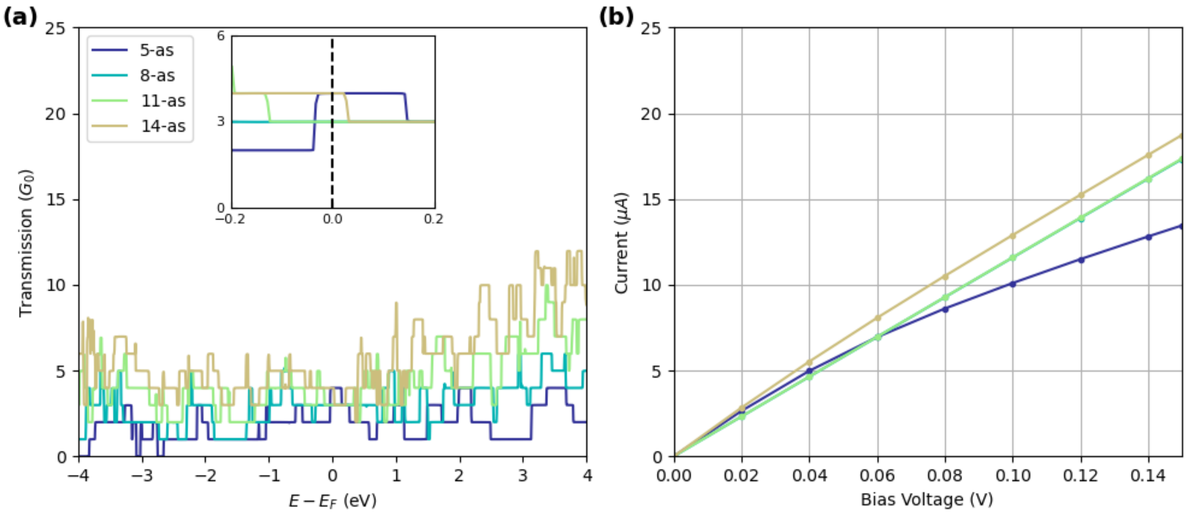

Fig S3: (a) Transmission at V=0 for various “as” edge nanoribbons. Inset shows transmission near E_F. (b) I-V characteristics of “as” edge nanoribbons.

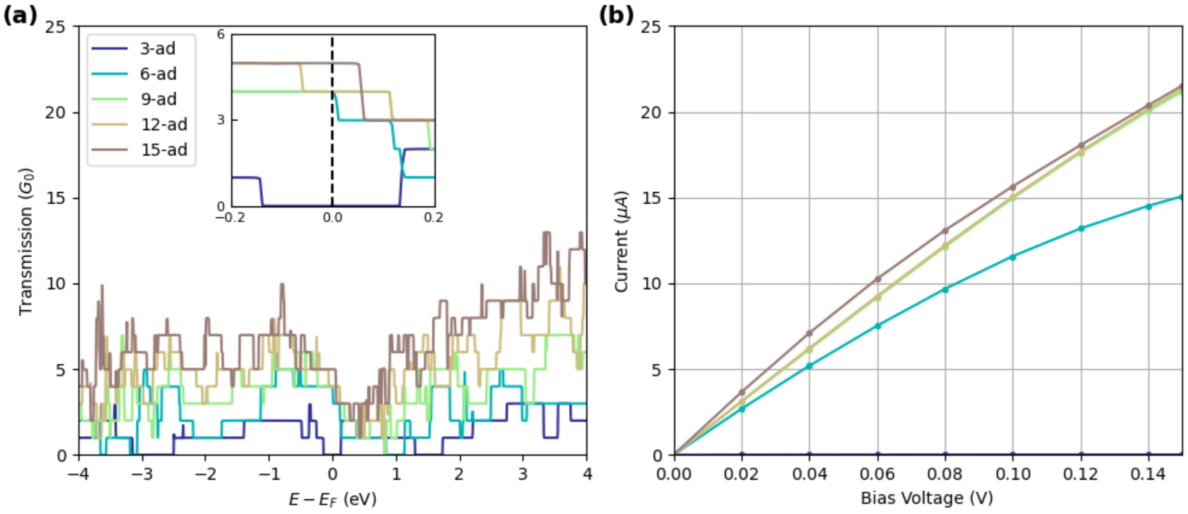

Fig S4: (a) Transmission at V=0 for various “ad” edge nanoribbons. Inset shows transmission near E_F. (b) I-V characteristics of “ad” edge nanoribbons.

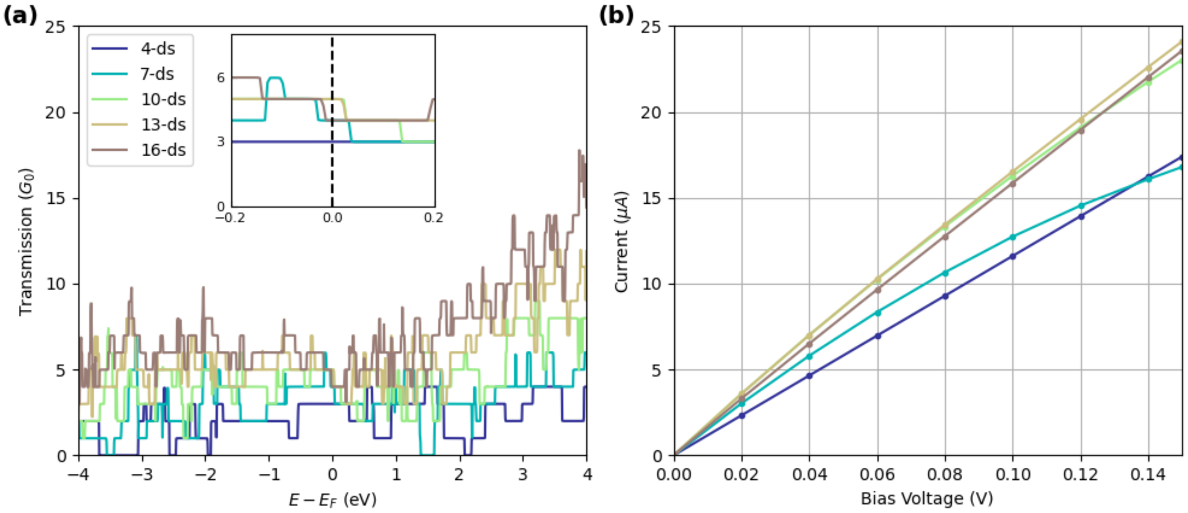

Fig S5: (a) Transmission at V=0 for various “ds” edge nanoribbons. Inset shows transmission near E_F. (b) I-V characteristics of “ds” edge nanoribbons.

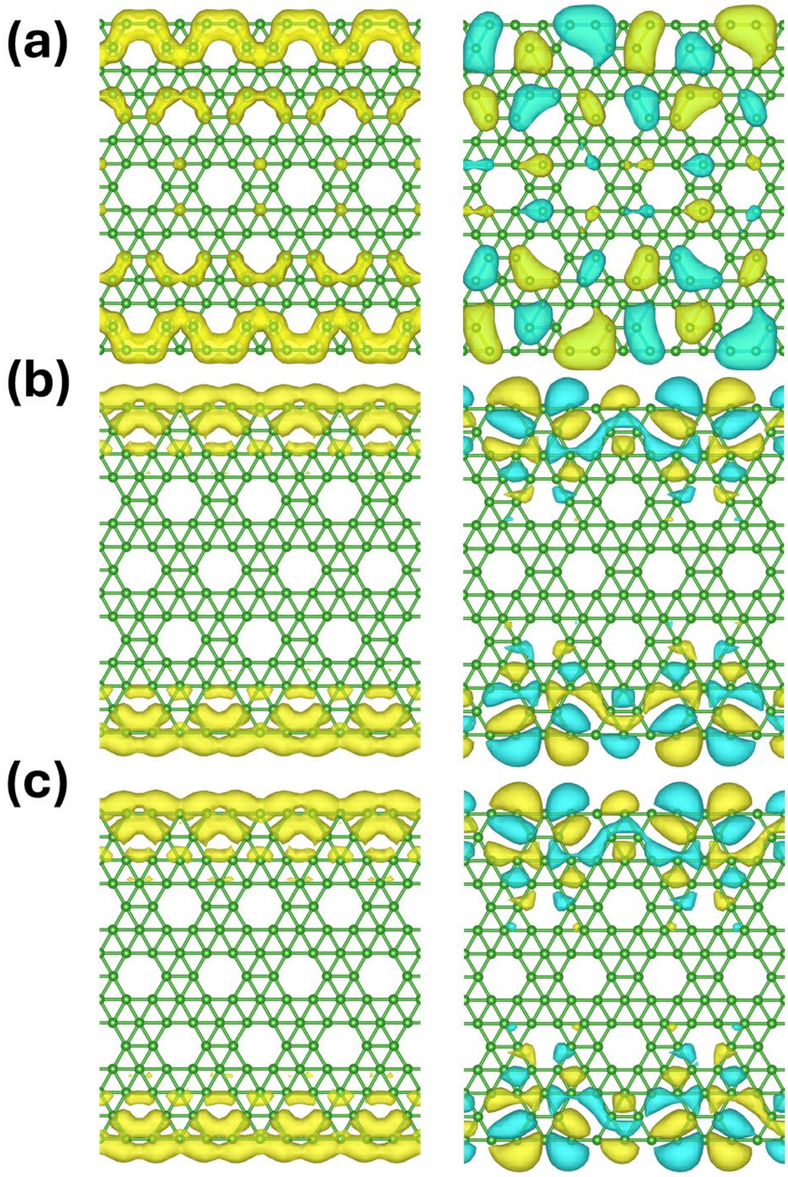

Fig S6: Edge localized transmission channels for “ss” edge nanoribbons shown as absolute (left) and real (right) parts of the wavefunction. Transmission channels (b) and (c) have identical absolute wavefunctions but their phases are symmetric/anti-symmetric across the ribbon width. When changing one edge, only one of (b) and (c) exists.
